# Enrichment of IFN-γ producing cells in different murine adipose tissue depots upon infection with an apicomplexan parasite

**DOI:** 10.1038/srep23475

**Published:** 2016-03-22

**Authors:** Luzia Teixeira, Raquel M. Marques, Pedro Ferreirinha, Filipa Bezerra, Joana Melo, João Moreira, Ana Pinto, Alexandra Correia, Paula G. Ferreira, Manuel Vilanova

**Affiliations:** 1 Departamento de Anatomia, ICBAS – Instituto de Ciências Biomédicas de Abel Salazar and UMIB – Unidade Multidisciplinar de Investigação Biomédica, Universidade do Porto, Rua de Jorge Viterbo Ferreira, 4050-313, Porto, Portugal; 2 Instituto de Investigação e Inovação em Saúde, Universidade do Porto, Portugal; IBMC–Instituto de Biologia Molecular e Celular, Universidade do Porto, 4200-135 Porto, Portugal; 3 Laboratório de Imunologia Mário Arala Chaves, ICBAS, Universidade do Porto.

## Abstract

Here we report that lean mice infected with the intracellular parasite *Neospora caninum* show a fast but sustained increase in the frequency of IFN-γ-producing cells noticeable in distinct adipose tissue depots. Moreover, IFN-γ-mediated immune memory could be evoked *in vitro* in parasite antigen-stimulated adipose tissue stromal vascular fraction cells collected from mice infected one year before. Innate or innate-like cells such as NK, NK T and TCRγδ^+^ cells, but also CD4^+^ and CD8^+^ TCRβ^+^ lymphocytes contributed to the IFN-γ production observed since day one of infection. This early cytokine production was largely abrogated in IL-12/IL23 p40-deficient mice. Moreover, production of IFN-γ by stromal vascular fraction cells isolated from these mice was markedly lower than that of wild-type counterparts upon stimulation with parasite antigen. In wild-type mice the increased IFN-γ production was concomitant with up-regulated expression of genes encoding interferon-inducible GTPases and nitric oxide synthase, which are important effector molecules in controlling intracellular parasite growth. This increased gene expression was markedly impaired in the p40-deficient mice. Overall, these results show that NK cells but also diverse T cell populations mediate a prompt and widespread production of IFN-γ in the adipose tissue of *N. caninum* infected mice.

The involvement of the adipose tissue in immune function has been increasingly recognized^1^,^2^. Indeed many cells of the immune system can be found in that tissue where some populations are enriched and/or display phenotypic characteristics distinct to those shown by cell counterparts present in lymphoid organs^3^,^4^,^5^,^6^. Since chronic low-grade adipose tissue inflammation has been associated with obesity-related diseases such as type 2 diabetes, many studies have addressed the immune components of adipose tissue in obese hosts^2^,^6^. However, the immune response to infection in the adipose tissue of lean hosts was studied in only a few reports that nevertheless showed that infections could have profound consequences in adipose tissue immune cell populations^7^,^8^,^9^,^10^,^11^,^12^,^13^,^14^,^15^. Increased numbers of macrophages and proinflammatory cytokines mRNA levels were observed in the adipose tissue of mice infected with diverse pathogenic agents such as adenovirus 36^10^, the bacterium *Yersinia pseudotuberculosis*^15^ and the protozoan parasites *Trypanosoma cruzi*^7^,^8^.

*N. caninum* is an intracellular protozoan parasite, closely related to *Toxoplasma gondii,* causative of clinical infections in diverse animal hosts occurring worldwide^16^. Neosporosis is particularly relevant in cattle where it is responsible for abortions causing heavy economic losses on dairy and beef industry^17^. Resistance against this infection has been associated with host production of pro-inflammatory cytokines IL-12 and IFN-γ. Genetic deficiencies for these cytokines^18^,^19^ or their neutralization upon specific mAb administration^20^,^21^ confer lethal susceptibility to neosporosis in otherwise resistant murine strains. Accordingly, IL-12Rβ2 chain-deficient mice but not wild-type (WT) counterparts are also highly susceptible to *N. caninum* infection^22^. In non-infected hosts, several lymphocytic populations have been shown to produce IFN-γ in the adipose tissue such as CD8^+^ T cells^23^; CD4^+^ T cells^24^,^25^; invariant natural killer T (NKT) cells^26^, γδT cells^27^ and natural killer (NK) cells^5^. Increased IFN-γ mRNA levels, indicative of a Th1-type immune response, were previously observed in the gonadal adipose tissue of *N. caninum* infected hosts^11^. Therefore, we aimed here at determining whether production of IFN-γ could be promoted upon infection in distinct adipose tissue depots and which cell types could be the source of this cytokine. The obtained results show that distinct lymphoid cell populations in both visceral and subcutaneous adipose tissue contribute to IFN-γ production and that local early production of this cytokine is largely dependent on IL-12/IL-23 p40. Moreover, and interestingly, they also show that parasite-specific memory as revealed by IFN-γ production is maintained in the adipose tissue at least for one year upon the infectious challenge.

## Results

### IFN-γ is early produced in the adipose tissue of mice challenged with *N. caninum* tachyzoites

**Figure 1:**
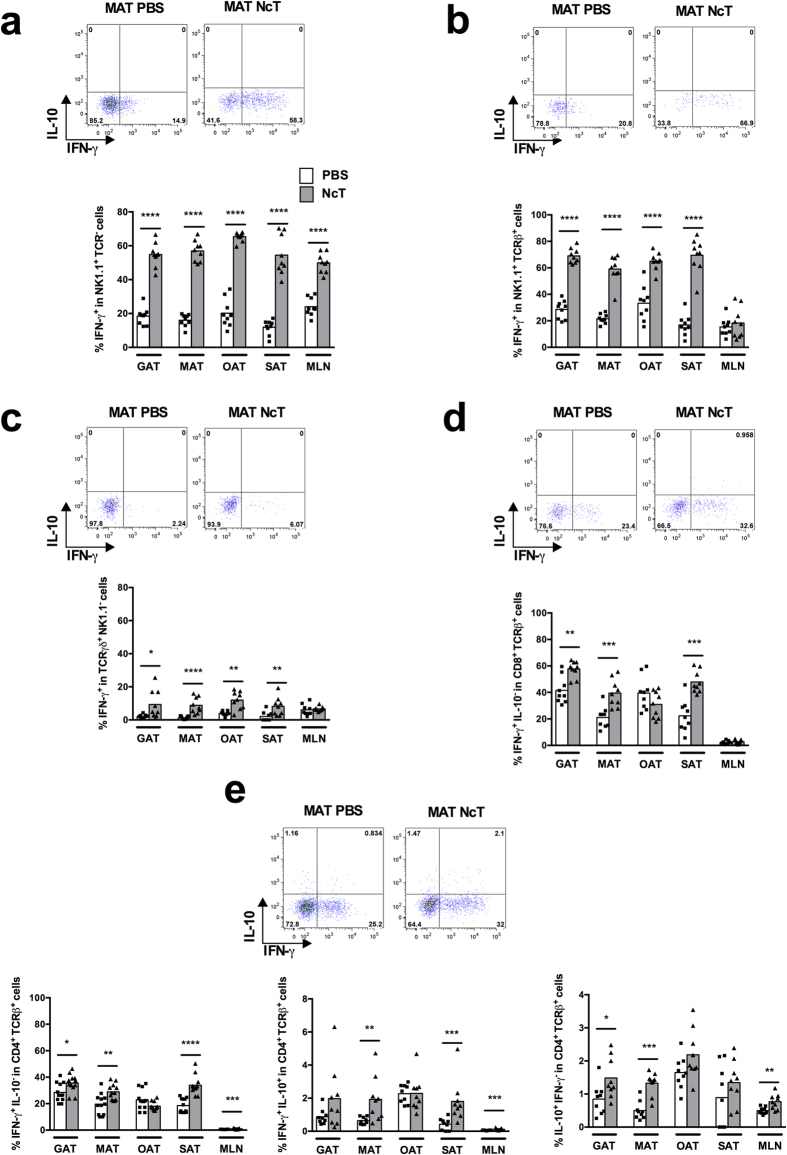
Prompt increase in the frequency IFN-γ^+^ cells in the adipose tissue of *N. caninum* infected mice. Frequencies of (**a**) IFN-γ^+^ NK1.1^+^ TCRβ^−^TCRγδ^−^ cells on total NK1.1^+^ cells, (**b**) IFN-γ^+^ NK1.1^+^ TCRβ^+^ TCRγδ^−^ cells on total NK1.1^+^ TCRβ^+^ cells, (**c**) IFN-γ^+^ TCRγδ^+^ NK1.1^−^ cells on total TCRγδ^+^ cells, (**d**) IFN-γ^+^ IL-10^−^CD8^+^ TCRβ^+^ TCRγδ^−^NK1.1^−^ cells on total CD8^+^ T cells and (**e**) IFN-γ^+^ IL-10^−^CD4^+^ TCRβ^+^ TCRγδ^−^NK1.1^−^, IFN-γ^+^ IL-10^+^ CD4^+^ TCRβ^+^ TCRγδ^−^NK1.1^−^ and IL-10^+^ IFN-γ^−^CD4^+^ TCRβ^+^ TCRγδ^−^NK1.1^−^ cells on total CD4^+^ T cells, in the gonadal, mesenteric, omental and subcutaneous adipose tissue (GAT, MAT, OAT and SAT, respectively) and mesenteric lymph nodes (MLN) from wild-type C57BL/6 mice sacrificed 24 h after intraperitoneal challenge with 1 × 10^7^ *N. caninum* tachyzoites (NcT) or PBS, as indicated. Each symbol represents an individual mouse. Bars represent means of 9 mice per group pooled from 3 independent experiments. Statistically significant differences between different experimental groups are indicated (Mann-Whitney U, *P < 0.05; **P ≤ 0.01; ***P ≤ 0.001; ****P ≤ 0.0001). Representative example of gating strategy used to define the respective cellular populations in the different depots of adipose tissue analysed. The example shown corresponds to MAT.

To determine which lymphoid populations could respond by producing IFN-γ in the adipose tissue of B6 mice infected intraperitoneally (i.p.) with *N. caninum*, we used flow cytometry and the gating strategy shown in Supplementary Fig. S1. The proportions of NK and NK T cells producing IFN-γ found were markedly increased as early as 24 h upon the parasitic challenge in all types of adipose tissue analysed (Fig. 1a,b). A slight increase in the frequency of IFN-γ^+^ TCRγδ^+^ cells was also observed (Fig. 1c). Interestingly, CD8^+^ TCRβ^+^ and CD4^+^ TCRβ^+^ cells were also found to be early producers of IFN-γ in the infected mice, as detected in most adipose tissue depots analysed (Fig. 1d,e and Supplementary Fig. S2). Contrastingly to this widespread cellular immune response detected in the gonadal, mesenteric and subcutaneous adipose tissue (GAT, MAT and SAT, respectively), only NK and CD4^+^ T cells produced IFN-γ in the mesenteric lymph nodes (MLN) of infected mice at frequencies higher than those detected in controls (Fig. 1a,e). CD4^+^ T cells simultaneously producing IL-10 and IFN-γ were also present at increased proportions in MAT, SAT and MLN of the *N. caninum* infected mice (Fig. 1e). Although early upon infection the proportions of IFN-γ-producing cells increased in all assessed populations, the numbers of NK cells producing IFN-γ per gram of adipose tissue were only found increased in SAT and those of TCR γδ^+^ cells in SAT, GAT and MAT (Supplementary Fig. S3). CD4^+^ T cells single producers of IL-10 were also detected at increased frequencies upon infection in GAT, MAT and MLN (Fig. 1e). Contrastingly, CD4^+^ T cells single producers of IL-4 were slightly decreased in frequency and number in the GAT from infected mice (Supplementary Fig. S2). Altogether these results show that in the adipose tissue of *N. caninum* infected mice a prompt production of the host protective cytokine IFN-γ occurs, which is mediated by NK cells but also by different T cell populations.

### Early production of IFN-γ in the adipose tissue of *N. caninum* infected mice is largely dependent on IL-12/IL-23p40

**Figure 2:**
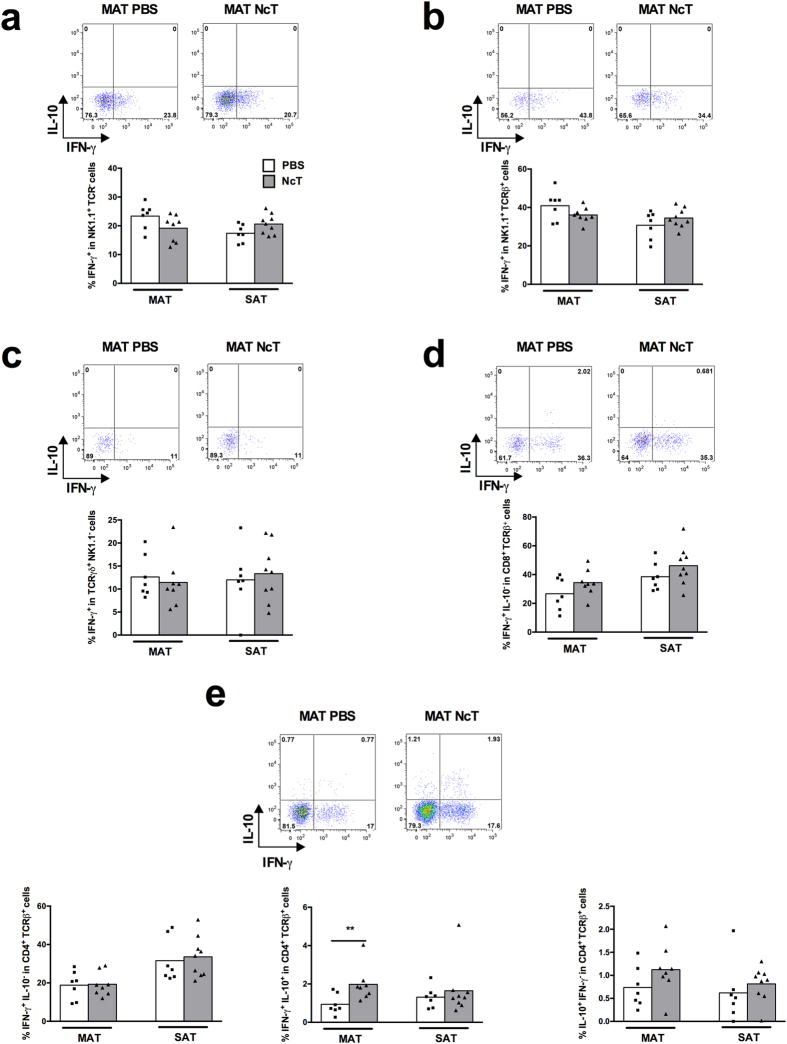
Impaired production of IFN-γ in the adipose tissue of infected IL-12/IL-23 p40^−/−^ mice. Frequencies of (**a**) IFN-γ^+^ NK1.1^+^ TCRβ^−^TCRγδ^−^ cells on total NK1.1^+^ cells, (**b**) IFN-γ^+^ NK1.1^+^ TCRβ^+^ TCRγδ^−^ cells on total NK1.1^+^ TCRβ^+^ cells, (**c**) IFN-γ^+^ TCRγδ^+^ NK1.1^−^ cells on total TCRγδ^+^ cells, (**d**) IFN-γ^+^ IL-10^−^CD8^+^ TCRβ^+^ TCRγδ^−^NK1.1^−^ cells on total CD8^+^ T cells and (**e**) IFN-γ^+^ IL-10^−^CD4^+^ TCRβ^+^ TCRγδ^−^NK1.1^−^, IFN-γ^+^ IL-10^+^ CD4^+^ TCRβ^+^ TCRγδ^−^NK1.1^−^ and IL-10^+^ IFN-γ^−^CD4^+^ TCRβ^+^ TCRγδ^−^NK1.1^−^ cells on total CD4^+^ T cells in the mesenteric and subcutaneous adipose tissue (MAT and SAT, respectively) from IL-12/IL-23 p40^−/−^ mice sacrificed 24 h after intraperitoneal challenge with 1 × 10^7^ *N. caninum* tachyzoites (NcT) or PBS, as indicated. Each symbol represents an individual mouse. Bars represent means of 7–9 mice per group pooled from 2 independent experiments. (Mann-Whitney U, **P ≤ 0.01). Representative example of gating strategy used to define the respective cellular populations in the different depots of adipose tissue analysed. The example shown corresponds to MAT.

**Figure 3:**
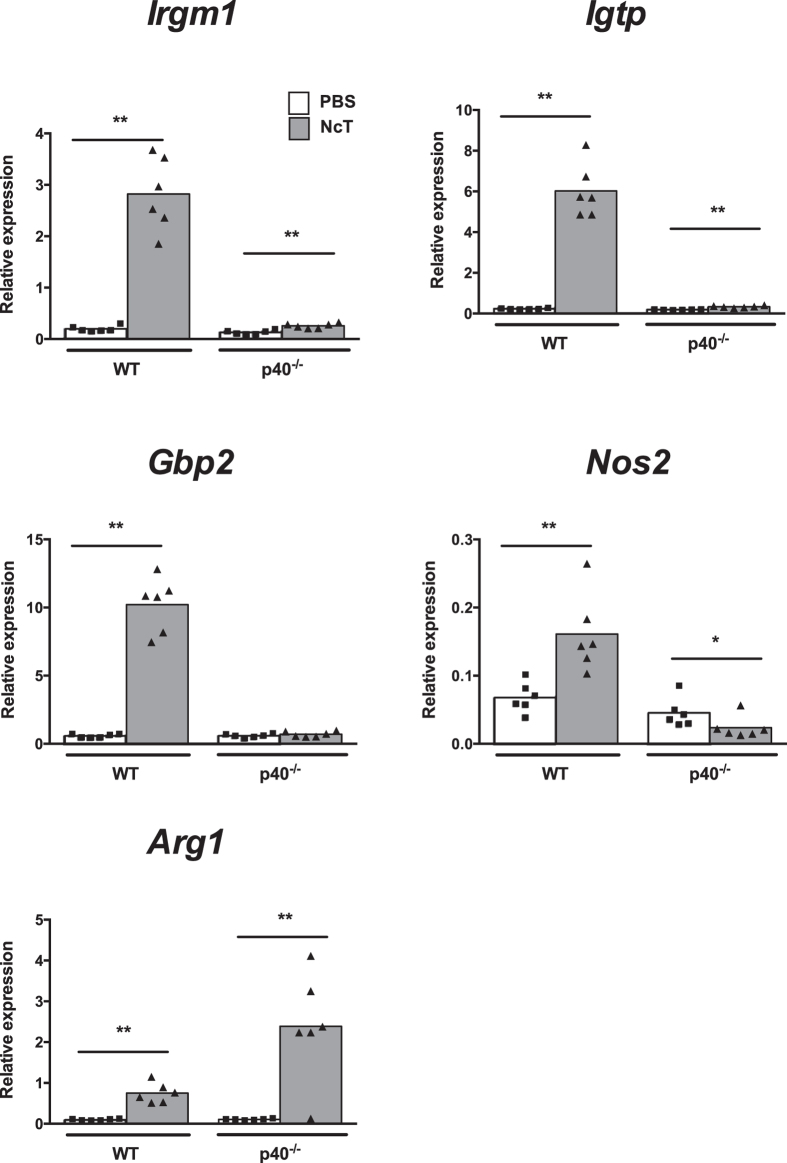
Increased expression of interferon-inducible GTPases and nitric oxide synthase 2 in the adipose tissue of infected mice. Relative levels of immunity-related GTPase family M member 1 (*Irgm1)*, interferon gamma induced GTPase *(Igtp)*, guanylate binding protein 2, interferon-inducible (*Gbp2),* nitric oxide synthase 2, inducible (*Nos2*) and *arginase* (*Arg1*) mRNA, normalized to Non-POU-domain containing octamer binding protein (*Nono*) mRNA, detected by real-time PCR in the SVF of mesenteric adipose tissue of wild-type (WT) and IL-12/IL-23 p40^−/−^ (p40^−/−^) mice 24 hours after intraperitoneal administration of 1 × 10^7^ *N. caninum* tachyzoites (NcT) or PBS. Each symbol represents an individual mouse. Bars represent means of 6 mice per group pooled from 2 independent experiments. (Mann-Whitney U, *P < 0.05; **P ≤ 0.01).

In contrast to what was observed in WT mice the proportions of cells producing IFN-γ were not found above those of sham-infected controls in the MAT and SAT of infected IL-12/IL-23 p40-deficient (p40^−/−^) mice except a detected increased frequency of IL-10 and IFN-γ double-producing CD4^+^ T cells in the MAT (Fig. 2). Therein and interestingly CD4^+^ T cells single producers of IL-4 concomitantly decreased in both frequency and number (Supplementary Fig. S2). The effector function of IFN-γ includes the up-regulation of genes encoding proteins involved in inhibition of intracellular parasite growth such as interferon-inducible GTPases and inducible nitric oxide synthase^28^. In the infected WT mice a 14-, 26-, 17- and 2-fold increase was respectively observed in immunity-related GTPase family M member 1 (*Irgm1*), interferon gamma induced GTPase (*Igtp*), guanylate binding protein 2 (*Gbp2*) and nitric oxide synthase 2, inducible (*Nos2*) mRNA levels, normalized to Non-POU-domain containing octamer binding protein (*Nono*) mRNA (Fig. 3). Similar results were obtained when normalized to hypoxanthine guanine phosphoribosyl transferase (*Hprt*) mRNA constitutive gene (data not shown). Contrastingly, a 1.9-fold decrease in *Nos2* mRNA levels and no change in *Gbp2* mRNA levels were detected in infected p40^−/−^ mice comparatively to non-infected counterparts upon normalization to *Nono* (Fig. 3) and to *Hprt* constitutive genes (data not shown). Nevertheless, a slight increase of *Irgm1* and *Igtp* mRNA expression (2- and 1.8-fold increase, respectively) was still observed in the infected p40^−/−^ mice comparatively to controls (Fig. 3). However, for *Igtp* this increase was only observed when *Nono* was used as reference gene as no difference was found between the two p40^−/−^ mouse groups when normalization was done to *Hprt* gene expression (Fig. 3 and data not shown). A marked increase (22-fold) in arginase 1 (*Arg1*) mRNA levels was observed in the infected highly susceptible p40^−/−^ mice comparatively to PBS-challenged controls whereas only a 7-fold increase was observed in infected WT mice (Fig. 3).

**Figure 4:**
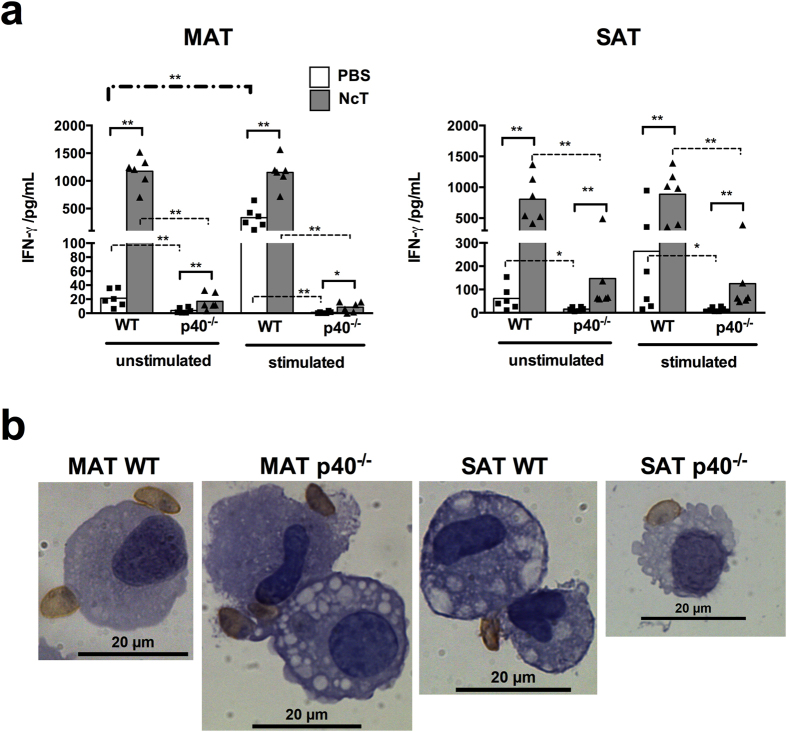
Impaired IFN-γ production by adipose tissue stromal vascular fraction cells isolated from infected IL-12/IL-23p40^−/−^ mice. (**a**) IFN-γ levels in the supernatants of mesenteric or subcutaneous adipose tissue (MAT and SAT, respectively) SVF cells cultured for 48 h alone (unstimulated) or in the presence of freeze-killed NcT (stimulated) recovered from control (PBS) or *N. caninum*-infected (NcT) wild type (WT) or IL-12/IL-23p40^−/−^ (p40^−/−^) C57BL/6 mice, as indicated, 24 h after intraperitoneal (i.p.) challenge. Each symbol represents an individual mouse. Bars represent means of 6 mice per group pooled from 2 independent experiments. (Mann-Whitney U, *P < 0.05; **P ≤ 0.01). (**b**) Representative images showing parasitic forms closely associated with stromal vascular fraction cells isolated from the MAT and SAT of WT or p40^−/−^ mice 24 h after i.p. administration of 1 × 10^7^ *N. caninum* tachyzoites, detected by immunohistochemistry. Cells were specific stained (brown coloration) with a polyclonal serum goat anti-*N. caninum* and counterstained with haematoxylin. This is one representative result of 2 independent experiments with 3 mice per group per experiment. Bar = 20 μm.

It was already shown that freeze-killed *Neospora caninum* tachyzoites (NcT) markedly induced *in vitro* IFN-γ production by murine spleen cells^29^. Similarly, adipose tissue stromal vascular fraction (SVF) cells isolated from MAT of PBS-treated WT mice responded to *in vitro* stimulation with freeze-killed *N. caninum* by producing IFN-γ (Fig. 4a). High levels of this cytokine were also detected in the culture supernatants of MAT and SAT SVF cells isolated from 24 h-infected WT mice without further stimulation (Fig. 4a). As NcT were observed in association with the SVF isolated cell samples (Fig. 4b), these parasites may provide the stimulus inducing the detected IFN-γ production in these cultures as well as in those of MAT and SAT cells of infected p40^−/−^ mice (Fig. 4a). Nevertheless, the levels of IFN-γ detected in culture supernatants of p40^−/−^ SVF cells were always lower than the ones detected in WT cell counterparts (Fig. 4a). Accordingly, upon *in vitro* stimulation with freeze-killed *N. caninum* no increased IFN-γ production was detected in the culture supernatants of MAT SVF cells isolated from p40^−/−^ mice (Fig. 4a). These results altogether indicate that adipose tissue resident cells have the capacity to promptly produce IFN-γ in response to *N. caninum* infection and show that this production is largely dependent on IL-12/IL-23 p40.

### Production of IFN-γ in the adipose tissue of *N. caninum* infected mice is sustainably increased

**Figure 5:**
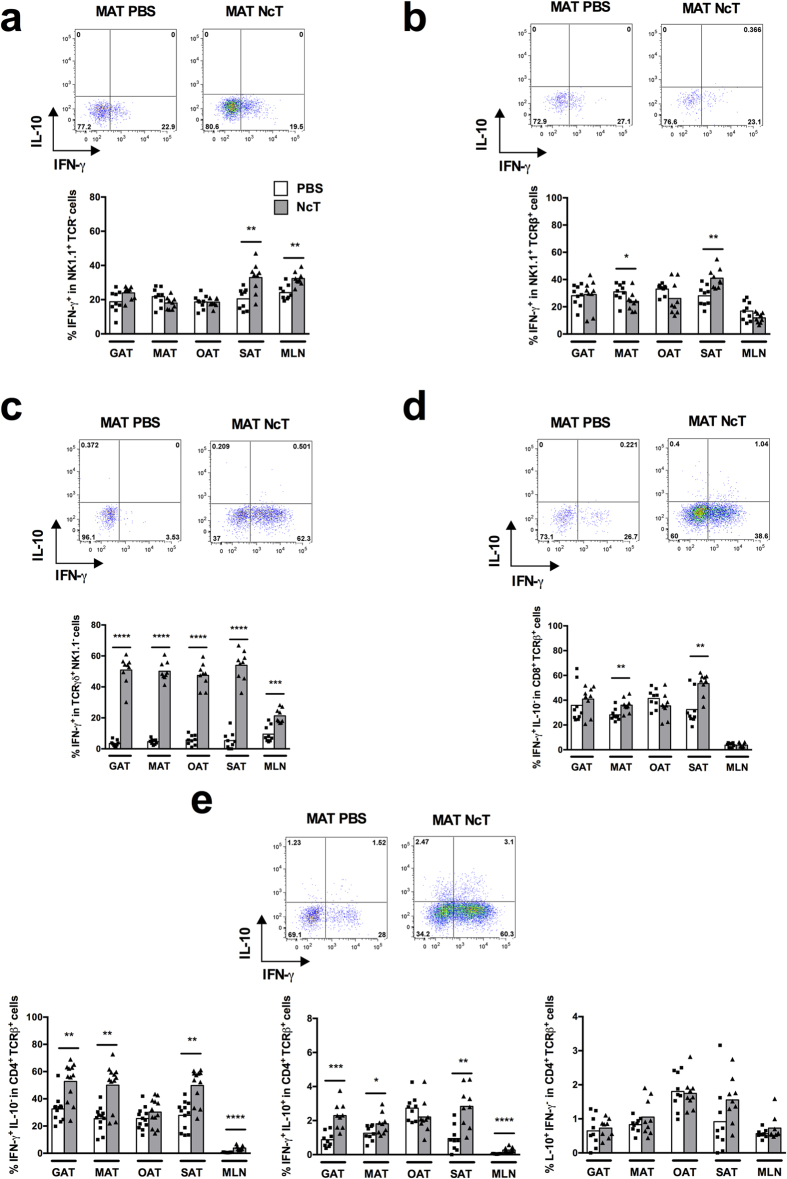
Sharp increase in the frequency of TCRγδ^+^ IFN-γ^+^ cells in the adipose tissue of *N. caninum* infected mice. Frequencies of (**a**) IFN-γ^+^ NK1.1^+^ TCRβ^−^TCRγδ^−^ cells on total NK1.1^+^ cells, (**b**) IFN-γ^+^ NK1.1^+^ TCRβ^+^ TCRγδ^−^ cells on total NK1.1^+^ TCRβ^+^ cells, (**c**) IFN-γ^+^ TCRγδ^+^ NK1.1^−^ cells on total TCRγδ^+^ cells, (**d**) IFN-γ^+^ CD8^+^ TCRβ^+^ TCRγδ^−^NK1.1^−^ cells on total CD8^+^ T cells and (**e**) IFN-γ^+ ^IL-10^−^CD4^+^ TCRβ^+^ TCRγδ^−^NK1.1^−^, IFN-γ^+^ IL-10^+^ CD4^+^ TCRβ^+^ TCRγδ^−^NK1.1^−^ and IL-10^+^ IFN-γ^−^CD4^+^ TCRβ^+^ TCRγδ^−^NK1.1^−^ cells on total CD4^+^ T cells in the gonadal, mesenteric, omental and subcutaneous adipose tissue (GAT, MAT, OAT and SAT, respectively) and mesenteric lymph nodes (MLN) observed 7 days after intraperitoneal challenge with 1 × 10^7^ *N. caninum* tachyzoites (NcT) or PBS, as indicated. Each symbol represents an individual mouse. Bars represent means of 9 mice per group pooled from 3 independent experiments. (Mann-Whitney U, *P < 0.05; **P ≤ 0.01; ***P ≤ 0.001; ****P ≤ 0.0001). Representative example of gating strategy used to define the respective cellular populations in the different depots of adipose tissue analysed. The example shown corresponds to MAT.

**Figure 6:**
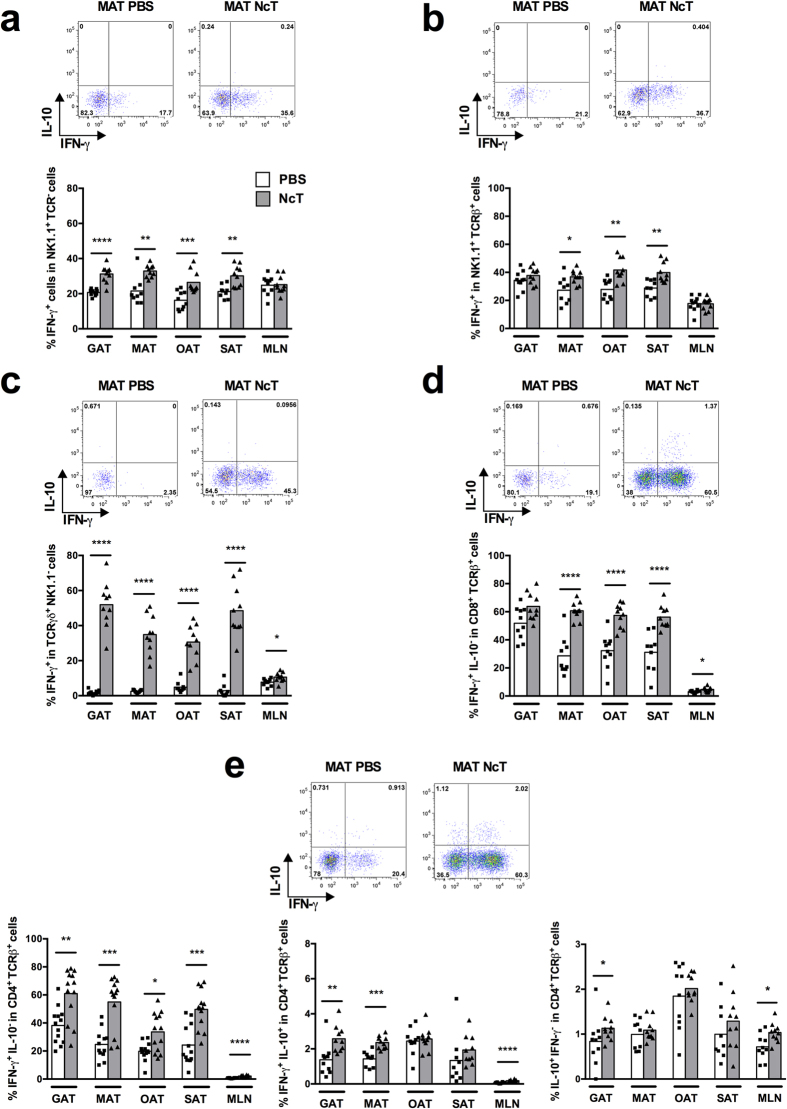
Sustained increase in the frequency of IFN-γ^+^ cells in the adipose tissue of *N. caninum* infected mice. Frequencies of (**a**) IFN-γ^+^ NK1.1^+^ TCRβ^−^TCRγδ^−^ cells on total NK1.1^+^ cells, (**b**) IFN-γ^+^ NK1.1^+^ TCRβ^+^ TCRγδ^−^ cells on total NK1.1^+^ TCRβ^+^ cells, **(c)** IFN-γ^+^ TCRγδ^+^ NK1.1^−^ cells on total TCRγδ^+^ cells, (**d**) IFN-γ^+^ CD8^+^ TCRβ^+^ TCRγδ^−^NK1.1^−^ cells on total CD8^+^ T cells and (**e**) IFN-γ^+^ IL-10^−^CD4^+^ TCRβ^+^ TCRγδ^−^NK1.1^−^, IFN-γ^+^ IL-10^+^ CD4^+^ TCRβ^+^ TCRγδ^−^NK1.1^−^ and IL-10^+^ IFN-γ^−^CD4^+^ TCRβ^+^ TCRγδ^−^NK1.1^−^ cells on total CD4^+^ T cells in the gonadal, mesenteric, omental and subcutaneous adipose tissue (GAT, MAT, OAT and SAT, respectively) and mesenteric lymph nodes (MLN) observed 21 days after intraperitoneal challenge with 1 × 10^7^ *N. caninum* tachyzoites (NcT) or PBS, as indicated. Each symbol represents an individual mouse. Bars represent means of 9 mice per group pooled from 3 independent experiments. (Mann-Whitney U, *P < 0.05; **P ≤ 0.01; ***P ≤ 0.001; ****P ≤ 0.0001). Representative example of gating strategy used to define the respective cellular populations in the different depots of adipose tissue analysed. The example shown corresponds to MAT.

Contrastingly to the observation made 24 h upon infection when a marked increase in the frequency of IFN-γ-producing NK and NK T cells was observed in all adipose tissue depots analysed, by 7 and 21 days this increase was slight and limited only to SAT in 7-day infected animals (Figs 5a,b and 6a,b). At this time point, the frequency of IFN-γ^+^ NK T cells actually decreased in the MAT (Fig. 5b). NK cells producing IFN-γ were also found increased in MLN by 7 days after the parasitic challenge while NK T cells did not respond in these lymphoid organs (Fig. 5a,b).

At day 7 upon infection a striking increase in the frequency of IFN-γ^+^ TCRγδ^+^ cells was observed in all adipose tissue depots analysed (Fig. 5c) that was still detected by 21 days after infection (Fig. 6c). T cells bearing the αβ TCR also responded by producing IFN-γ in the infected animals with IFN-γ^+^ CD8^+^ and IFN-γ^+^ CD4^+^ T cells reaching proportions similar to those detected for γδ T cells (Figs 5c–e and 6c–e). Nevertheless, in the non-infected controls high proportions of αβ T cells either CD4^+^ or CD8^+^ producing IFN-γ were already detected (Figs 5d and 6d), that were higher than those found in the MLN of control mice (p < 0.0001 when comparing MLN vs GAT, MAT, OAT and SAT at any time point analysed, n = 9/group/time point). In agreement, others have reported a high frequency of IFN-γ-expressing T cells in visceral adipose tissue of lean hosts^25^. The frequency of IFN-γ^+^ CD4^+^ TCRβ^+^ cells increased upon infection in all depots analysed 7 days after infection, except OAT, and was still above controls by 21 days after infection (Figs 5e and 6e). Contrastingly, the proportions of IL-4-producing CD4^+^ T cells decreased in the GAT and MAT 7 and 21 days after infection and also in SAT in the later time-point (Supplementary Fig. S5). On the other hand, the frequency of IL-4 and IFN-γ double producers increased in MAT and OAT 7 and 21 days after infection and also in SAT 21 days after infection (Supplementary Fig. S5). The frequency of IL-10 and IFN-γ double producers within CD4^+^ T cells also increased 7 days upon infection in all tissues analysed, except OAT, and was still detected increased in GAT and MAT by 21 days (Figs 5e and 6e). IFN-γ^+^ CD8^+^ TCRβ^+^ cells were also found at increased proportions in MAT and SAT 7 and 21 days after infection and also in OAT in the later time point (Figs 5d and 6d). Altogether these results show that *N. caninum* infection induced a marked response by T cells in the adipose tissue that is biased towards the production of IFN-γ, a host protective cytokine in this infection.

**Figure 7:**
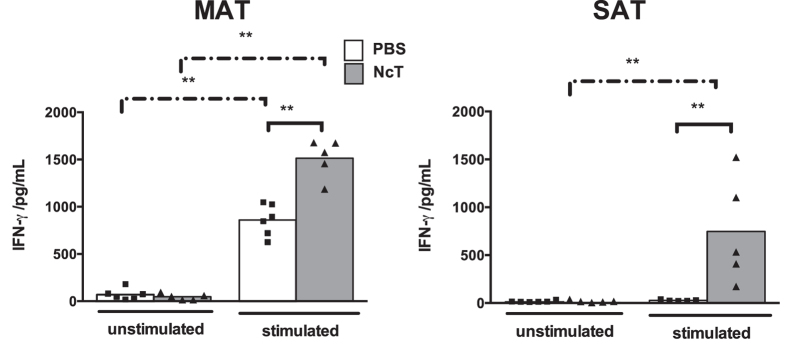
IFN-γ-mediated long-term memory in adipose tissue of *N. caninum* infected mice. IFN-γ levels in the supernatants of mesenteric and subcutaneous adipose tissue (MAT and SAT, respectively) stromal vascular fraction cells cultured for 48 h alone (unstimulated) or in the presence of freeze-killed NcT (stimulated) recovered from control (PBS) or *N. caninum*-infected wild-type (NcT) mice, as indicated, one year after challenge. Each symbol represents an individual mouse. Bars represent means of 5–6 mice per group pooled from 2 independent experiments. (Mann-Whitney U, **P ≤ 0.01).

Having observed that in the first weeks upon infection adipose tissue T cells predominantly produced IFN-γ, we further determined whether this response could lead to antigen-specific memory in this tissue. Therefore, we isolated SVF cells from the MAT and SAT of infected mice one year after the i.p. parasitic challenge and stimulated them *in vitro* with freeze-killed NcT. As shown in Fig. 7, higher levels of IFN-γ were detected in culture supernatants of killed-NcT-stimulated SVF cells isolated from MAT and SAT of infected mice as compared to those of controls from non-infected mice. To determine if one year after the parasitic challenge mice were still infected and to evaluate the possibility of infection recrudescence, the parasitic burden was determined in the lungs, a major target organ in acute neosporosis^30^, GAT, that was previously shown to be transiently colonized after a similar i.p. infection^11^, and brain, a target organ for chronic *N. caninum* persistence^30^. Parasitic DNA was detected in the brain of 2 out of 5 infected animals whereas no parasitic DNA was detected in GAT or lungs indicating that although a chronic infection was established, the parasite did not reactivate. Altogether, these results show that parasite-specific long-term immune memory was maintained in the adipose tissue even in the absence of detectable local parasite colonization.

## Discussion

In this work a prompt but sustained increase in IFN-γ-producing cells was observed in distinct adipose tissue depots of mice infected with *N. caninum*. Bovine NK cells displayed increased IFN-γ production upon *in vitro* stimulation with *N. caninum*-infected bovine fibroblasts or with NcT^31^. Accordingly, in the infected mice higher proportions of IFN-γ-producing NK cells were observed in adipose tissue, concomitant with parasite detection. NK T cells were also early stimulated to produce IFN-γ in the adipose tissue of distinct anatomical locations. This effect was not observed in the MLN in accordance with the previous remarked particular characteristics of NK T cells present in adipose tissue^3^. Interestingly, CD4^+^ and CD8^+^ αβ T cells were also early producers of IFN-γ in the infected mice. As the adipose tissue naturally presents a high frequency of T cells displaying a memory phenotype^32^,^33^, this likely explains the prompt production of IFN-γ upon *N. caninum* infection. A previous report has shown that memory CD8^+^ T cells produce IFN-γ early upon infection in an antigen-independent manner, in response to IL-12 and IL-18^34^. Moreover, *in vitro* studies showed that CD8^+^ T cells isolated from the adipose tissue of lean mice produce IFN-γ in response to IL-12 and IL-18 alone^23^. As in p40^−/−^ mice the increase in adipose tissue IFN-γ^+^ CD8^+^ T cells proportions elicited by infection was abrogated it would be worth determining if it could depend on IL-12. The IL-12/IL-23 p40-dependent early production of IFN-γ in the adipose tissue was not confined to the CD8^+^ T cell population as it was also abrogated for NK T, TCRγδ^+^, and CD4^+^ T cells in infected p40^−/−^ mice. In murine listeriosis, splenic NK1.1^+^ cells, CD8^+^ and CD4^+^ T cells were also shown to be early sources of IFN-γ^35^. Nevertheless, a slight increase in the proportions of CD4^+^ T cells simultaneously producing IFN-γ and IL-10 was still observed in *N. caninum* infected p40^−/−^ mice. Interestingly in *T. gondii* infected hosts, a population of IFN-γ and IL-10 double-producing CD4^+^ T cells was shown to better control the replication of parasites inside macrophages than single IFN-γ producers^36^. Therefore the increased frequency of CD4^+^ IFN-γ^+^ IL-10^+^ T cells detected in the adipose tissue of infected p40^−/−^ mice may nevertheless contribute to some control of local parasitic replication. In accordance with the increased proportions of these cells detected in the infected p40^−/−^ mice, slightly increased levels of IFN-γ were detected in the culture supernatants of SVF cells isolated from MAT and SAT of infected p40^−/−^ mice. This increase was however much lower than the one observed in similar cultures of cells obtained from infected WT mice, in accordance with the previously described impaired capacity of p40^−/−^ mice to produce IFN-γ upon antigenic stimulation^37^. Moreover, freeze-killed NcT induced *in vitro* the production of IFN-γ by MAT SVF cells of WT control mice while such effect was not induced in SVF cells of p40^−/−^ mice further reinforcing the idea that the production of IFN-γ induced by *N. caninum* in the adipose tissue is mainly IL-12/IL-23 p40 dependent. Whether this effect could be mediated by IL-12, IL-23, p40 monomer or other putative heterodimer that can be formed with extracellular p40 monomer^38^ remains to be determined. As we detected these differences in IFN-γ production, we assessed whether this would translate into different expression levels of genes regulated by this cytokine, such as the ones encoding immunity-related GTPases (IRGs) and guanylate-binding proteins (GBPs). These proteins are important for destruction of the parasitophorous vacuole in cells infected by the *N. caninum* related protozoan *T. gondii*^28^. Increased expression levels of interferon-inducible GTPases mRNA were previously detected in the brain^39^,^40^ and spleen^40^ of *N. caninum* infected mice. In accordance, we observed here an up-regulated expression of *Irgm1*, *Igtp* and *Gbp2* in the adipose tissue early upon infection. IRGM1, IGTP and GBP2 have been shown to inhibit *T. gondii* replication in macrophages^41^,^42^. Therefore, a similar effect may also take place in the adipose tissue of *N. caninum* infected mice. Indeed, in p40^−/−^ mice, where IRGs and GBP2 gene expression was only marginally up-regulated, a heavy parasitic colonization in the adipose tissue was previously observed 7 days after infection^11^. *Nos2* expression, indicative of M1 type macrophage polarization, was found up-regulated in WT mice early upon infection whereas it was down-regulated in p40^−/−^ mice. *In vitr*o studies have shown that NO production by peritoneal macrophages induced by IFN-γ inhibits parasitic multiplication^43^. All these effector mechanisms that are down regulated or only slightly increased in the infected p40^−/−^ mice can contribute to an impaired control of parasite replication locally in the adipose tissue. Moreover, *Arg1* expression, that was found markedly up-regulated in the infected p40^−/−^ mice, has been associated with increased host susceptibility in infections caused by other intracellular pathogens^44^.

TCRγδ^+^ cells have been shown to mediate host protection in other parasitic infections^45^,^46^. As the population of γδT cells was the only one consistently showing elevated proportions of IFN-γ^+^ cells in all adipose tissue depots and time points analysed upon infection it would be worth exploring its role in the course of *N. caninum* infection. Others have already implicated γδ T cells in host defensive mechanisms against this parasite in the bovine host^47^. CD4^+^ T cells, also implicated in host resistance to neosporosis^48^, were found to be producing IFN-γ sustainably in the adipose tissue upon *N. caninum* infection. Using OVA-specific OT-II mice, others have shown that adipose tissue SVF macrophages can promote IFN-γ production by CD4^+^ T cells^33^. As macrophages were found at increased numbers in the adipose tissue of *N. caninum* infected mice^11^, it would be interesting to determine whether these leukocyte cells could be promoting local lymphocyte IFN-γ production observed therein. Indeed, a previous report showed that *N. caninum-*challenged bovine macrophages can promote IFN-γ production by CD4^+^ T cells^49^. T cells simultaneously producing IFN-γ and IL-10 were found to increase in different adipose tissue depots of the infected mice. This IL-10 production by these cells can be important to prevent IFN-γ mediated-immunopathology as described in *T. gondii* infection^36^.

Helminthic parasites have been shown to promote Th2-type responses in the adipose tissue^12^,^13^. In *N. caninum* infected mice increased splenic mRNA and serum levels of the Th2-type signature cytokine IL-4 were previously observed^50^,^51^. In the adipose tissue a decreased frequency of IL-4 single producer CD4^+^ T cells concomitant with increased proportions of CD4^+^ T cells producing both IFN-γ and IL-4 was found. Memory Th2 cells may acquire expression of IFN-γ when primed in conditions promoting Th1 development^52^. A similar phenomenon may occur in the *N. caninum* infected mice as the majority of resident T cells in the adipose tissue already present an effector-memory phenotype^32^,^33^. A distinct splenic T cell population producing both IL-4 and IFN-γ has been also described in mice infected with helminth parasites, which induce marked Th2-type responses^53^,^54^. Similar double producers were found here in the context of a parasitic infection that induced a marked Th1 bias. It would be worth determining in future studies whether the concomitant IL-4 and IFN-γ production could be a mechanism limiting an excessive Th1-type immune response.

A role in initiating and maintaining adipose tissue inflammation has been previously suggested for CD8^+^ T cells^55^. CD8^+^ T cells were shown to promptly respond by producing IFN-γ in the intestinal mucosa of *N. caninum*-infected mice^56^. Here we also showed a prompt but persistent increase in the frequency of CD8^+^ T cells producing IFN-γ in the adipose tissue of the infected mice that was more marked by 21 days of infection than at previous time points. As IFN-γ produced by CD8^+^ T cells was shown to play a significant host protective role in neosporosis^40^, this population may contribute to local protection against this parasite. A persistently increased frequency of CD8^+^ T cells producing IFN-γ was also recently reported in VAT of mice infected with *Listeria monocytogenes*^12^. Others have shown the presence of CD8^+^ memory T cells in fat pad up to 59 and 296 days after infection with *L. monocytogenes* and vesicular stomatitis virus, respectively^57^. Moreover, increased proportions of activated CD8^+^ T cells, as well as of CD4^+^ T cells, were observed 15 months upon infection in the adipose tissue of virally infected hosts^14^. We show here that MAT and SAT SVF cells isolated from mice one year after infection was established produce high levels of IFN-γ upon *in vitro* parasite antigen-recall indicating that memory cells can persist in these tissues in the long-term and are responsive to *N. caninum.*

The majority of the studies addressing the host immunity to *N. caninum* focused on the immune response occurring in lymphoid organs. Here, we have addressed the immune response to *N. caninum* infection occurring in a non-lymphoid tissue and have shown that upon the parasitic challenge an IFN-γ-mediated response is fast and concomitantly elicited in both visceral and subcutaneous adipose tissue. Moreover we have identified NK cells as well as TCRγδ^+^ cells and distinct TCRβ^+^ cell populations as cell sources of this host protective cytokine. Altogether, our results highlight the involvement of the adipose tissue in the host protective immune response to *N. caninum*.

## Methods

### Mice

Female WT B6 mice (7–8 week old) were purchased from Charles River and kept at the animal facilities of the Institute of Biomedical Sciences Abel Salazar (Porto, Portugal) during the experiments. IL-12/IL-23 p40-deficient B6 mice were purchased from Jackson Laboratories (Bar Harbor, Maine, USA) and housed and bred also at ICBAS in individual ventilated cages. Hiding and nesting materials were provided. Procedures involving mice were performed according to the European Convention for the Protection of Vertebrate Animals used for Experimental and Other Scientific Purposes (ETS 123) and directive 2010/63/EU of the European parliament and of the council of 22 September 2010 on the protection of the animals used for scientific purposes, and Portuguese rules (DL 113/2013). Authorization to perform the experiments was issued by competent national board authority, Direcção-Geral de Alimentação e Veterinária (0420/000/000/2012 and 0421/000/000/2015).

### Parasites

NcT (Nc-1, ATCC® [50843]) were obtained as previously described^51^. As the virulence of *N. caninum* is attenuated if maintained for a long time in tissue culture^58^, in all our experiments the parasites used underwent <15 *in vitro* passages from the original ATCC vial. The viability of the used inocula was confirmed in highly susceptible p40^−/−^ mice^19^. Others have shown that 2 h freezing at −70 °C was enough to inactivate NcT^29^. Therefore, for preparation of freeze-killed NcT, suspensions of live tachyzoites (prepared as described above) were centrifuged at 1500 × g for 15 min at 6 °C, the supernatant discarded and the NcT containing-pellet kept at least four days frozen at −80 °C. On the day of the experiment, the pellet was resuspended in RPMI-1640 medium supplemented with 10 mM Hepes, 85 IU/ml penicillin, 85 μg/ml streptomycin, 62,5 ng/mL of amphotericin B, 50 μM 2-mercaptoethanol (all from all from Sigma-Aldrich, St Louis, USA) and 10% FBS (Gibco, MA, USA) (complete RPMI) by passing through a syringe with a 25G needle and applied to the cell cultures.

### Challenge infections

*N. caninum* infections were performed in 8–20 weeks-old female WT or p40^−/−^ B6 mice by the i.p. route, by inoculation of 0.5 ml PBS containing 1 × 10^7^ tachyzoites. Mock-infected controls were similarly i.p. injected with 0.5 ml of PBS.

### Collection of biological samples

Twenty-four hours, 7 and 21 days and 12 months after infection, mice were isoflurane anesthetized for retro-orbital blood collection and euthanized by cervical dislocation. For flow cytometry analysis, GAT (VAT present in broad ligament of uterus and ovaries), MAT (VAT between the two peritoneal layers of the mesentery), OAT (VAT associated to the greater omentum; in the dissection, pancreas was carefully avoided), inguinal SAT (carefully avoiding inguinal lymph nodes) and MLN were removed and placed in Hanks’s balanced salt solution supplemented with 4% bovine serum albumin (BSA) and 10 mM Hepes Buffer (all from Sigma-Aldrich) for further analysis. In the one-year experiments, GAT, lungs and brain were collected from all mice and stored at −80 °C for DNA extraction.

### Isolation of stromal vascular fraction cells

SVF cells were isolated as previously described in detail^11^. Briefly, after collagenase II digestion, samples were homogenized to single-cell suspensions, passed through a 100 μm cell strainer and centrifuged at 280 × g for 10 min at 4 °C. Cells at the bottom, corresponding to the SVF were resuspended in complete RPMI medium for 48 h SVF cell cultures or in RPMI −1640 supplemented with 10 mM Hepes, 60 IU/ml penicillin, 60 μg/ml streptomycin, 50 μM 2-mercaptoethanol, and 10% FBS for SVF cell cultures to be used in flow cytometric analysis.

### Flow cytometric analysis

For cytokine intracellular staining, SVF cells (1 × 10^6^cells per well) isolated as described above were incubated for 4 h 30 min at 37 °C with 500 ng/mL of ionomycin, 50 ng/mL PMA and 10 μg/mL of Brefeldin A (all from Sigma). Cells were pre-incubated with anti-mouse CD16/CD32 (clone 93) followed by surface staining with FITC anti-mouse TCRγδ (clone GL3), APC anti-mouse NK1.1 (clone PK136), APC-eFluor^®^ 780 anti-mouse CD8 (clone 53-6.7), eFluor® 450 anti-mouse TCRβ (clone H57-597) (all from eBioscience, San Diego, CA) and Brilliant Violet 510^TM^ anti-mouse CD4 (clone RM4-5) (BioLegend, San Diego, CA). Cells were then fixed with 2% formaldehyde, washed, permeabilized with 0.5% saponin (Sigma) and pre-incubated with anti-mouse CD16/CD32 (clone 93) before intracellular staining with PE anti-mouse IL-10 (clone JES5-16E3), PerCP-Cyanine5.5 anti-mouse IFN-γ (clone XMG1.2) and PE-Cy7 anti-mouse IL-4 (clone 11B11) or respective isotype controls (PE Rat IgG2b, k (clone eB149/10H5); PerCP-Cyanine5.5 Rat IgG1 Isotype Control (clone eBRG1) and PE-Cyanine7 Rat IgG1 K (clone eBRG1).

Data acquisition was performed on a FACSCanto™ II system (BD Biosciences, San Jose, CA) using the FACSDiva™ software (BD) and compensated and analysed in FlowJo version 9.7.5. (Tree Star, Inc., Ashland, OR). A biexponential transformation was applied to improve data visualization. Fluorescence minus one (FMO) gating was used to define the gates for IL-10^+^, IFN-γ^+^ and IL-4^+^ cells. Isotype controls were used to evaluate unspecific staining. Due to the high interference of PercpCy5.5 in the channel detecting PE-Cy7 and to assure that detection of IFN-γ and IL-4 double production CD4^+^ T cells was not an artefact, in some experiments the same cells were also stained with FITC anti-mouse IFN-γ (clone XMG1.2)(BD Biosciences) instead of PerCP-Cyanine5.5 anti-mouse IFN-γ and no antibody was added in the PercpCy5.5 channel (Supplementary Fig. S1c,d). By using this staining, similar frequencies of IFN-γ and IL-4 double producer cells were obtained thus validating the presented results.

### IFN-γ detection in culture supernatants of SVF cells

WT and p40^−/−^ MAT and SAT SVF cells were added to 96 well plates (3,5 × 10^5^ SVF cells/well) and cultured for 48 h in complete RMPI alone or with 1,75 × 10^6^ freeze-killed NcT at a ratio cell:NcT of 1:5, prepared as described above. IFN-γ levels in culture supernatants were quantified with Ready-Set-Go!® ELISA (eBioscience) according to manufacturer’s instructions.

### Cytospin immunohistochemistry

Cytospins of SVF cells isolated from MAT and SAT of mice sacrificed 24 h after infection with *N. caninum* were prepared as follows. The slides were methanol fixed and specifically stained for *N. caninum* by a previously described protocol^11^. Briefly, peroxidase activity was blocked by treatment with 0.3% hydrogen peroxide in methanol (Merck, Darmstadt, Germany) for 20 min. Sections were then incubated in a moist chamber for 20 min with normal rabbit serum (Dako, Glostrup, Denmark) diluted 1:5 in 10% BSA (Sigma), to eliminate non-specific staining. Excess serum was removed and the sections were incubated at room temperature, 1h45 min with goat anti-*N. caninum* polyclonal serum (VMRD, Pullman, WA) diluted 1:1500. Sections incubated with anti-*N. caninum* antibody were washed and incubated for 30 min at room temperature with the peroxidase-labeled rabbit anti-goat secondary antibody (Millipore, Billerica, MA, USA) diluted 1:500. The colour in all sections was developed by incubation with 3,3′-diaminobenzidine (Dako). After counterstaining tissue sections with Mayer’s Haematoxylin (Merck), slides were mounted in Entellan (Merck). A positive reaction was indicated by the presence of brown cytoplasmic staining.

### PCR for the detection of NcT

DNA from the brain, lungs, and GAT of infected and PBS-treated mice, or from NcT to use as positive standards, was extracted and *N. caninum* DNA was detected as previously described in detail^11^. DNA samples corresponding to 10^3^ to 10^0^ NcT were included as external standards.

### RNA isolation and real time PCR analysis

Total RNA extraction (from 10^6^ MAT SVF cells of WT and p40^−/−^ mice) and cDNA synthesis were performed as previously described in detail^11^. Real-time PCR was then used for the semi-quantification of *Irgm1*, *Igtp*, *Gbp2*, *Nos2 and Arg1* mRNA expression levels with the Kapa SYBR Fast qPCR Kit (Kapa Biosystems Inc, Wilmington, MA) in a Rotor-Gene 6000 (Corbett life science, Sydney, Australia). As reference genes we used *Hprt* and *Nono*. For the quantification of mRNA expression levels, the reaction was performed in a final volume of 10 μL containing 0.2 μM of each specific primer^11^,^40^: *Nono* forward: GCTCTTTTCTCGGGACGG, *Nono* reverse: GCATTTTTGTACCCTTGACTT GGA; *Hprt* forward: ACATTGTGGCCCTCTGTGTG, *Hprt* reverse: TTATGTCCCCCGTTGACTGA, *Irgm1* forward: CTCTGGATCAGGGTTTGAGGAGTA; *Irgm1* reverse: GGAACTGTGTGATGG TTTCATGATA; *Gbp2* forward: TGAGTACCTGGAACATTCACTGAC; *Gbp2* reverse: AGTCGCGGCTCATTAAAGC; *Igtp* forward: CTGAGCCTGGATTGCAGCTT; *Igtp* reverse: GTCTATGTCTGTGGGCCTGA; *Arg1* forward: CTCCAAGCCAAAGTCCTTA GAG; *Arg1* reverse: AGGAGCTGTCATTAGGGACATC; *Nos2* forward: CCAAGCCCT CACCTACTTCC; *Nos2* reverse: CTCTGAGGGCTGACACAAGG (all from Tib Molbiol, Berlin, Germany) and 1 × Master Mix plus 1 μL of the newly-synthesized cDNA. The PCR program run was as follows: 1) denaturation at 95 °C, 5 min 2) amplification in 35 cycles (95 °C, 10 s; 62 °C, 20 s). We analysed real-time PCR data by the comparative threshold cycle (C_T_) method^59^. Individual relative gene expression values were calculated using the following formula: 2 ^− (C_T_ gene of interest − C_T_ constitutive gene)^^59^.

### Statistical analysis

Statistical significance of results was determined by non-parametric Mann-Whitney U test calculated with GraphPad Prism 6.0 software. (*P ≤ 0.05; **P ≤ 0.01; ***P ≤ 0.001; ****P ≤ 0.0001). The data presented is from 2 to 3 pooled independent experiments with n = 6–9 mice/group as indicated in respective figure legends. Each individual mouse is represented in figures by a symbol and bars represent means of each experimental group.

## Additional Information

**How to cite this article**: Teixeira, L. *et al.* Enrichment of IFN-γ producing cells in different murine adipose tissue depots upon infection with an apicomplexan parasite. *Sci. Rep.* **6**, 23475; doi: 10.1038/srep23475 (2016).

## Supplementary Material

Supplementary Information
