# Supplementary material for: Enrichment of IFN-γ producing cells in different murine adipose tissue depots upon infection with an apicomplexan parasite
Source: Sci Rep. 2016 Mar 22;6:23475. doi: 10.1038/srep23475 (PMC4802212; doi:10.1038/srep23475)
Supplement: Supplementary Information [file srep23475-s1.pdf]

Enrichment of IFN-γ producing cells in different murine adipose tissue depots upon infection with an apicomplexan parasite

Luzia Teixeira[1*], Raquel M Marques[1,+], Pedro Ferreirinha[2,3,+], Filipa Bezerra[1], Joana Melo[1], João Moreira[1], Ana Pinto[1], Alexandra Correia[2], Paula G Ferreira[1], Manuel Vilanova[2,3]

[1]Departamento de Anatomia, ICBAS – Instituto de Ciências Biomédicas de Abel Salazar and UMIB – Unidade Multidisciplinar de Investigação Biomédica, Universidade do Porto, Rua de Jorge Viterbo Ferreira, 4050-313, Porto, Portugal. [2]Instituto de Investigação e Inovação em Saúde, Universidade do Porto, Portugal; IBMC – Instituto de Biologia Molecular e Celular, Universidade do Porto, 4200-135 Porto, Portugal. [3]Laboratório de Imunologia Mário Arala Chaves, ICBAS, Universidade do Porto.

[+]these authors contributed equally to this work

*lmteixeira@icbas.up.pt

Supplementary Figure S1: Gating strategy to define immune cellular populations

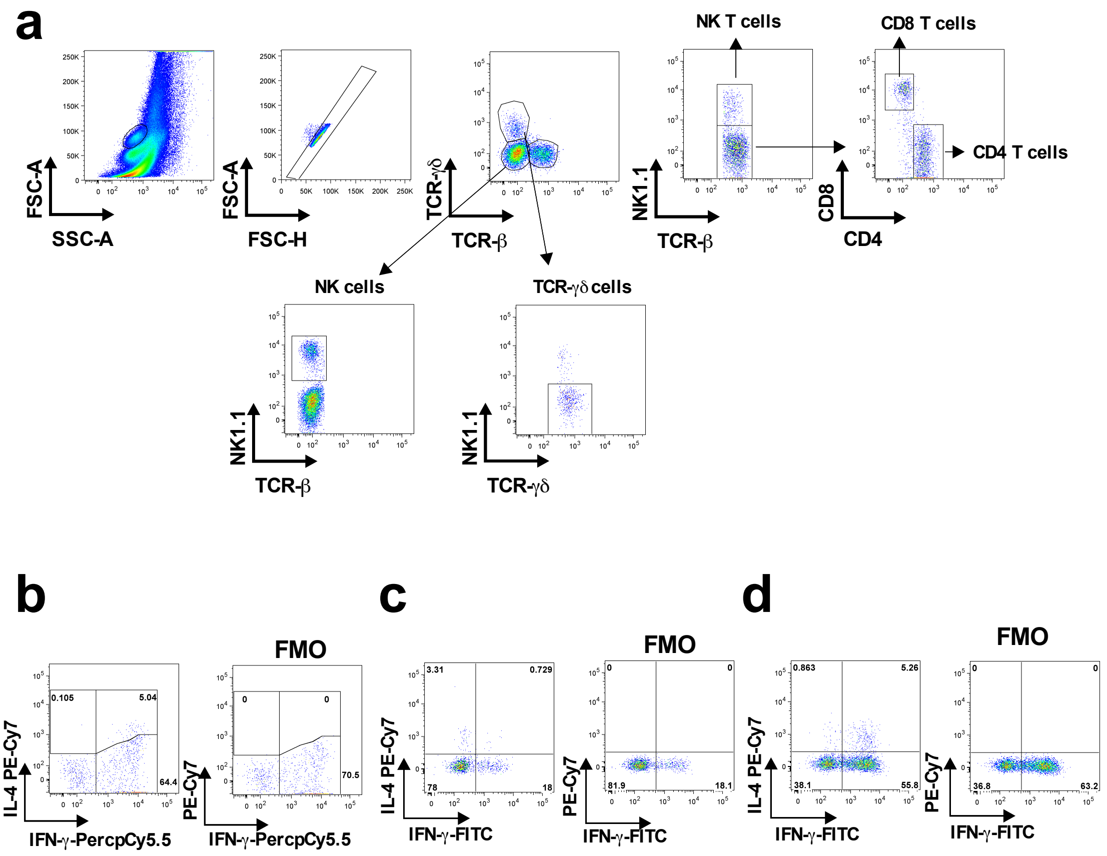

a) Flow cytometry gating strategy used to define NK, NK T, TCRγδ, CD8 T and CD4 T cells in the stromal vascular fraction (SVF) of the different depots of adipose tissue analysed. A gate for lymphocytes was selected and singlets were then selected from FSC-A versus FSC-H dot plot. NK cells were defined as NK1.1$^+$TCRβ$^-$TCRγδ$^-$, NK T cells as NK1.1$^+$TCRβ$^+$TCRγδ$^-$, gamma delta T cells as TCRγδ$^+$NK1.1$^-$, CD4 T cells as CD4$^+$TCRβ$^+$NK1.1$^-$TCRγδ$^-$, CD8 T cells as CD8$^+$TCRβ$^+$NK1.1$^-$TCRγδ$^-$. Dot plots are representative examples using SVF cells isolated from mesenteric adipose tissue (MAT) of mice 21 days after intraperitoneal challenge with PBS. **b)** Representative dot plots of IL4$^+$IFN-γ$^-$, IL-4$^+$IFN-γ$^+$ and IL4$^-$IFN-γ$^+$ CD4$^+$ T cells SVF cells, gated in CD4$^+$TCRβ$^+$NK1.1$^-$TCRγδ$^-$, from subcutaneous adipose tissue of mice, 21 days after *N. caninum* infection. The fluorescence minus one (FMO) control for IL-4 is also shown. Representative dot plots of IL4$^+$IFN-γ$^-$, IL-4$^+$IFN-γ$^+$ and IL4$^-$IFN-γ$^+$ CD4$^+$ T cells in SVF cells, gated in CD4$^+$TCRβ$^+$NK1.1$^-$TCRγδ$^-$ cells, from MAT of mice 21 days after **c)** PBS or **d)** parasitic challenge using IFN-γ FITC. The respective fluorescence minus one (FMO) control for IL-4 is also shown.

Supplementary Figure S2: Early decrease in frequency and number of IL-4-producing T cells in adipose tissue of infected mice.

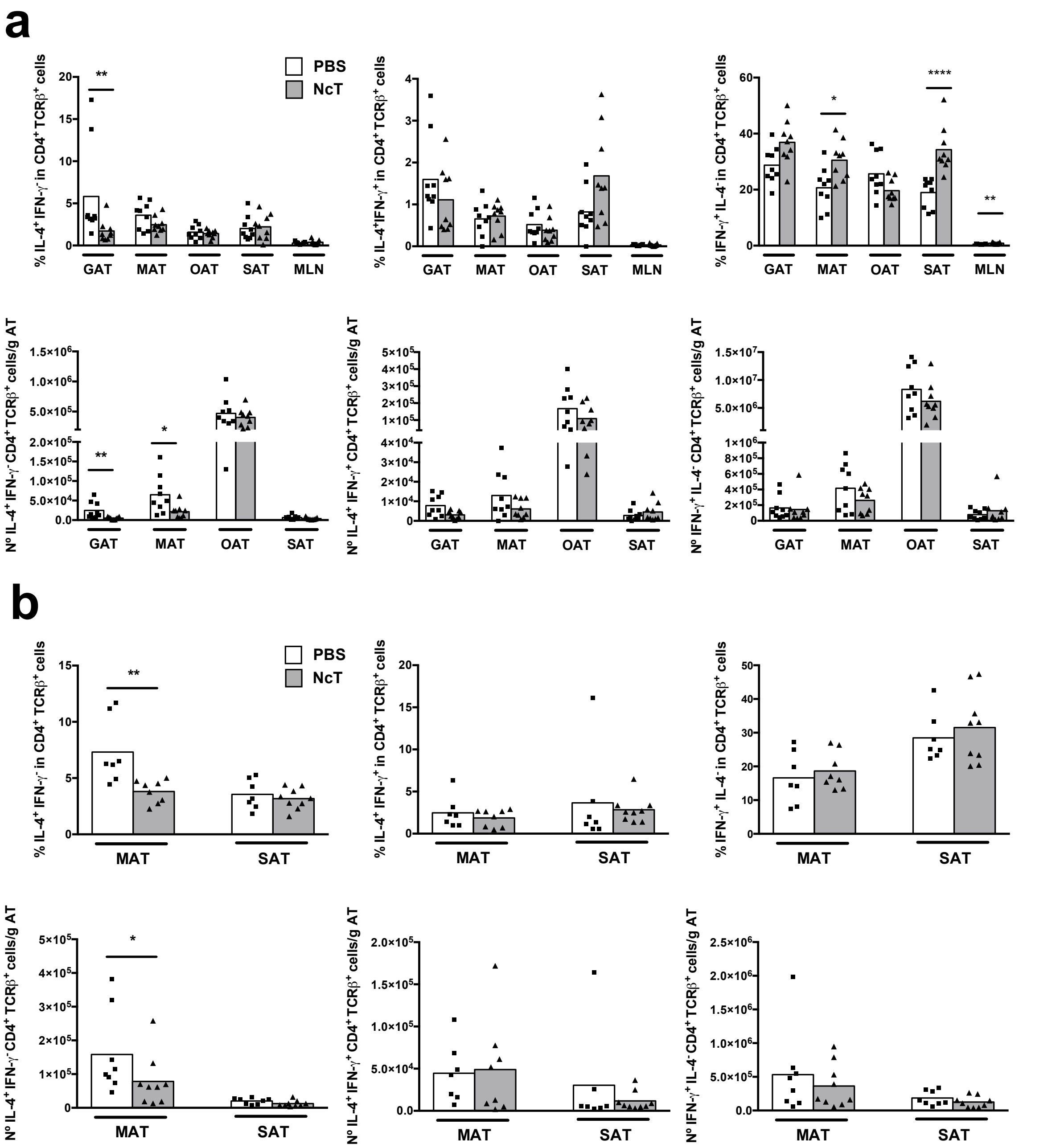

Frequency of IL-4^{+}IFN-γ^{-}CD4^{+}, IL-4^{+}IFN-γ^{+}CD4^{+}, IL-4^{-}IFN-γ^{+}CD4^{+} cells on total CD4^{+}TCRβ^{+}TCR$\gamma\delta^{-}$NK1.1^{-} cells in the gonadal, mesenteric, omental and subcutaneous adipose tissue (GAT, MAT, OAT and SAT, respectively) and mesenteric lymph nodes (MLN) at 24h after intraperitoneal challenge with 1×10^{7} *N. caninum* tachyzoites (NcT) or PBS, as indicated of **(a)** wild-type mice or **(b)** IL-12/IL-23p40$^{-/-}$ mice. Numbers per gram of adipose tissue of the same cellular populations are also shown from **(a)** wild-type mice or **(b)** IL-12/IL-23p40$^{-/-}$ mice for the indicated tissues. Each symbol represents an individual mouse. Bars represent means of **(a)** 9 mice per group pooled from 3 independent experiments or **(b)** 7-9 mice per group pooled from 2 independent experiments. Statistically significant differences between different experimental groups are indicated (Mann-Whitney U test, *$P < 0.05$; **$P \leq 0.01$; ****$P \leq 0.0001$).

Supplementary Figure S3: Increased numbers of IFN-γ-producing cells in the adipose tissue of *Neospora caninum*-infected mice.

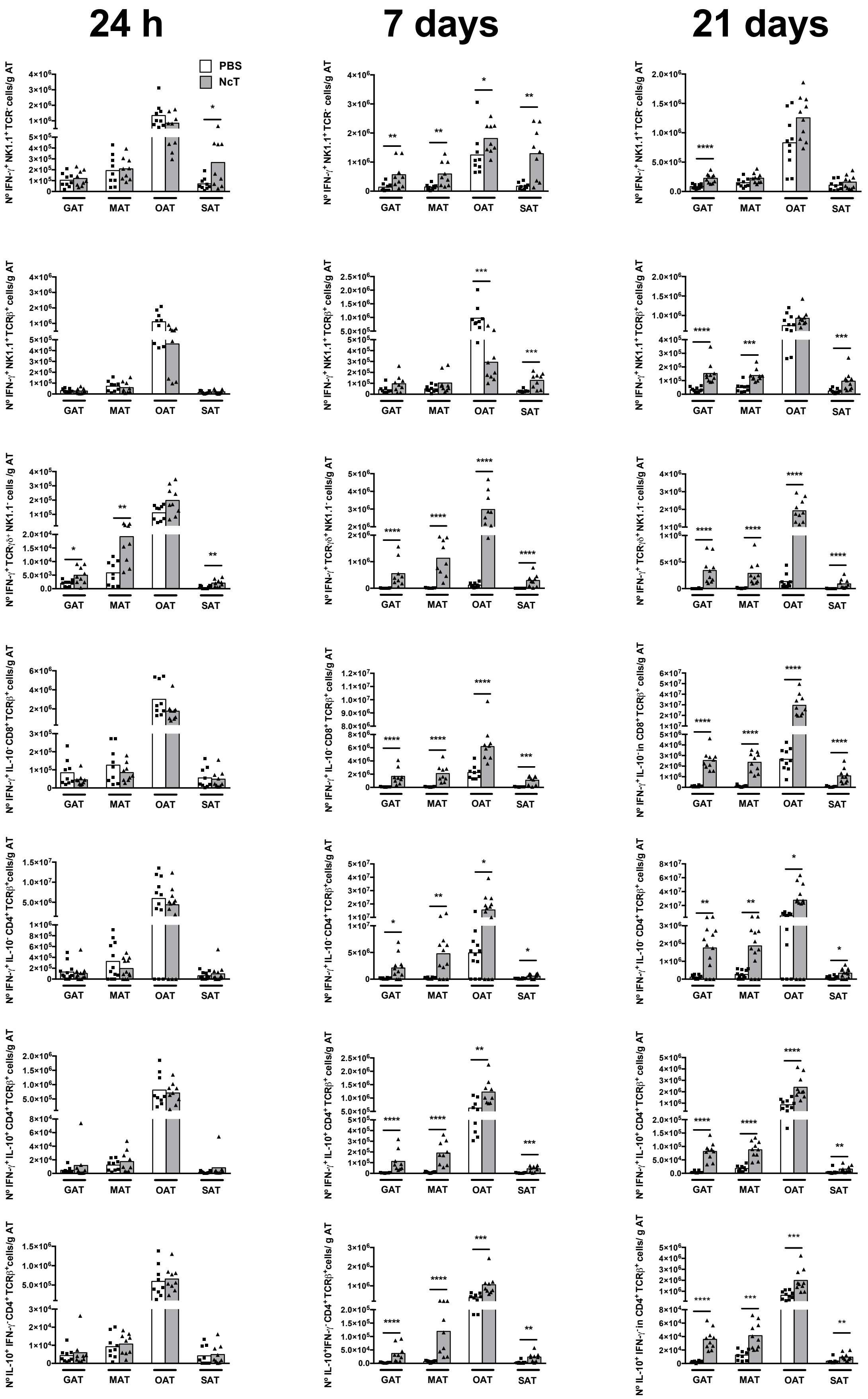

Numbers of IFN-γ^+NK1.1$^+$TCRβ^-TCR$\gamma\delta^-$ cells, IFN-γ^+NK1.1$^+$TCR-β^+TCR$\gamma\delta^-$ cells, IFN-γ^+TCR-$\gamma\delta^+$NK1.1$^-$ cells, IFN-γ^+CD8$^+$TCRβ^+TCR$\gamma\delta^-$NK1.1$^-$ cells, IFN-γ^+IL-10$^-$CD4$^+$TCRβ^+TCR$\gamma\delta^-$NK1.1$^-$ cells, IFN-γ^+IL-10$^+$CD4$^+$TCRβ^+TCR$\gamma\delta^-$NK1.1$^-$ cells, and IL-10$^+$IFN-γ^-CD4$^+$TCRβ^+TCR$\gamma\delta^-$NK1.1$^-$ cells per gram of adipose tissue in the gonadal, mesenteric, omental and subcutaneous adipose tissue (GAT, MAT, OAT and SAT, respectively) at 24h, 7 and 21 days after intraperitoneal challenge with $1{\times}10^7$ *N. caninum* tachyzoites (NcT) or PBS, as indicated. Each symbol represents an individual mouse. Bars represent means of 9 mice per group pooled from 3 independent experiments. Statistically significant differences between different experimental groups are indicated (Mann-Whitney U test, *$P < 0.05$; **$P \leq 0.01$; ***$P \leq 0.001$; ****$P \leq 0.0001$).

Supplementary Figure S4: No change in the numbers of IFN-γ-producing cells in the adipose tissue of IL-12/IL-23p40$^{-/-}$ mice upon *N. caninum* infection.

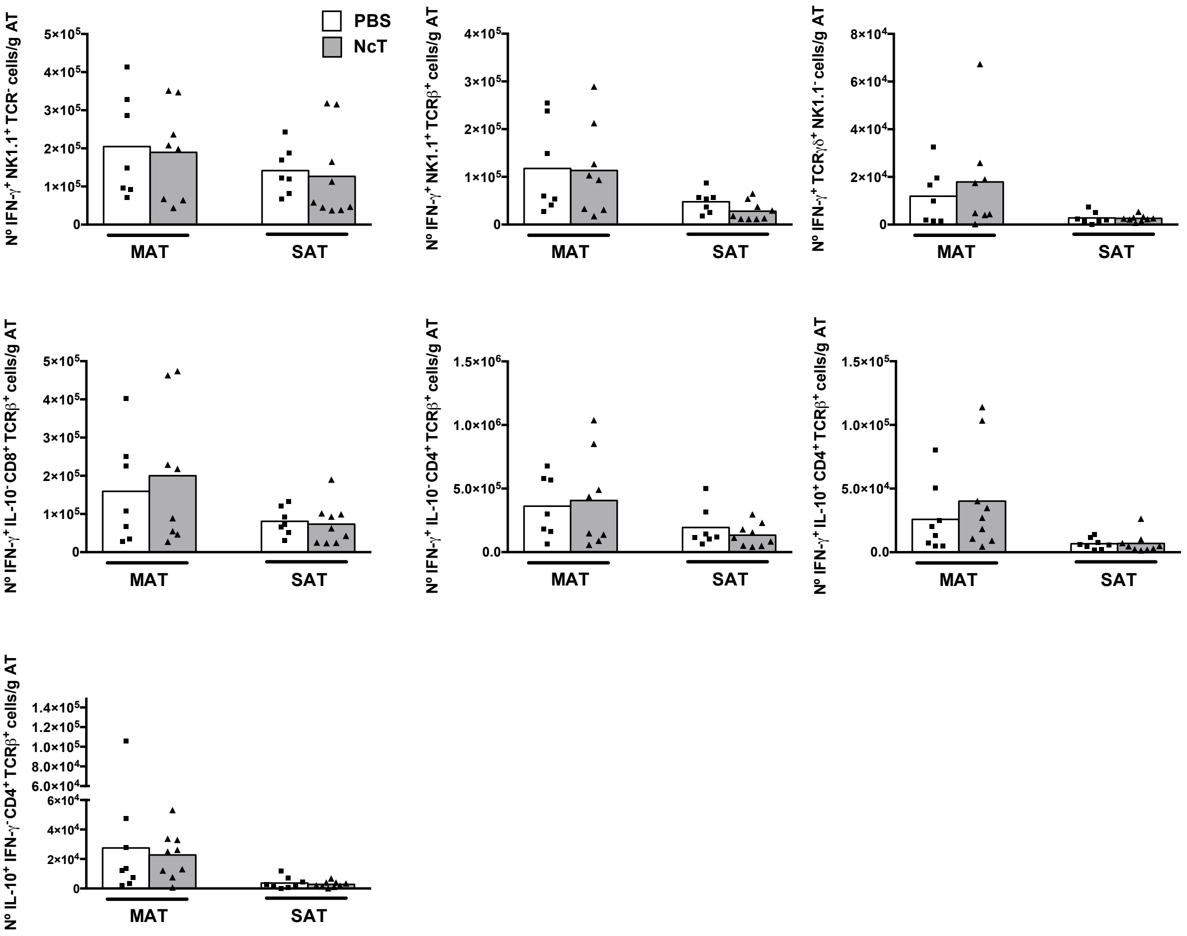

Numbers of IFN-γ^+NK1.1$^+$TCRβ^-TCR$\gamma\delta^-$ cells, IFN-γ^+NK1.1$^+$TCR-β^+TCR$\gamma\delta^-$ cells, IFN-γ^+TCR-$\gamma\delta^+$NK1.1$^-$ cells, IFN-γ^+CD8$^+$TCRβ^+TCR$\gamma\delta^-$NK1.1$^-$ cells, IFN-γ^+IL-10$^-$CD4$^+$TCRβ^+TCR$\gamma\delta^-$NK1.1$^-$ cells, IFN-γ^+IL-10$^+$CD4$^+$TCRβ^+TCR$\gamma\delta^-$NK1.1$^-$ cells, and IL-10$^+$IFN-γ^-CD4$^+$TCRβ^+TCR$\gamma\delta^-$NK1.1$^-$ cells per gram of adipose tissue in the mesenteric and subcutaneous adipose tissue (MAT and SAT, respectively) at 24h after intraperitoneal challenge with 1×10^7 *N. caninum* tachyzoites (NcT) or PBS, as indicated. Each symbol represents an individual mouse. Bars represent means of 7-9 mice per group pooled from 2 independent experiments. No statistically significant differences between different experimental groups were found (Mann-Whitney U test).

Supplementary Figure S5. Sustained increase in number and frequency of IL-4 and IFN-γ double producers upon *N. caninum* infection.

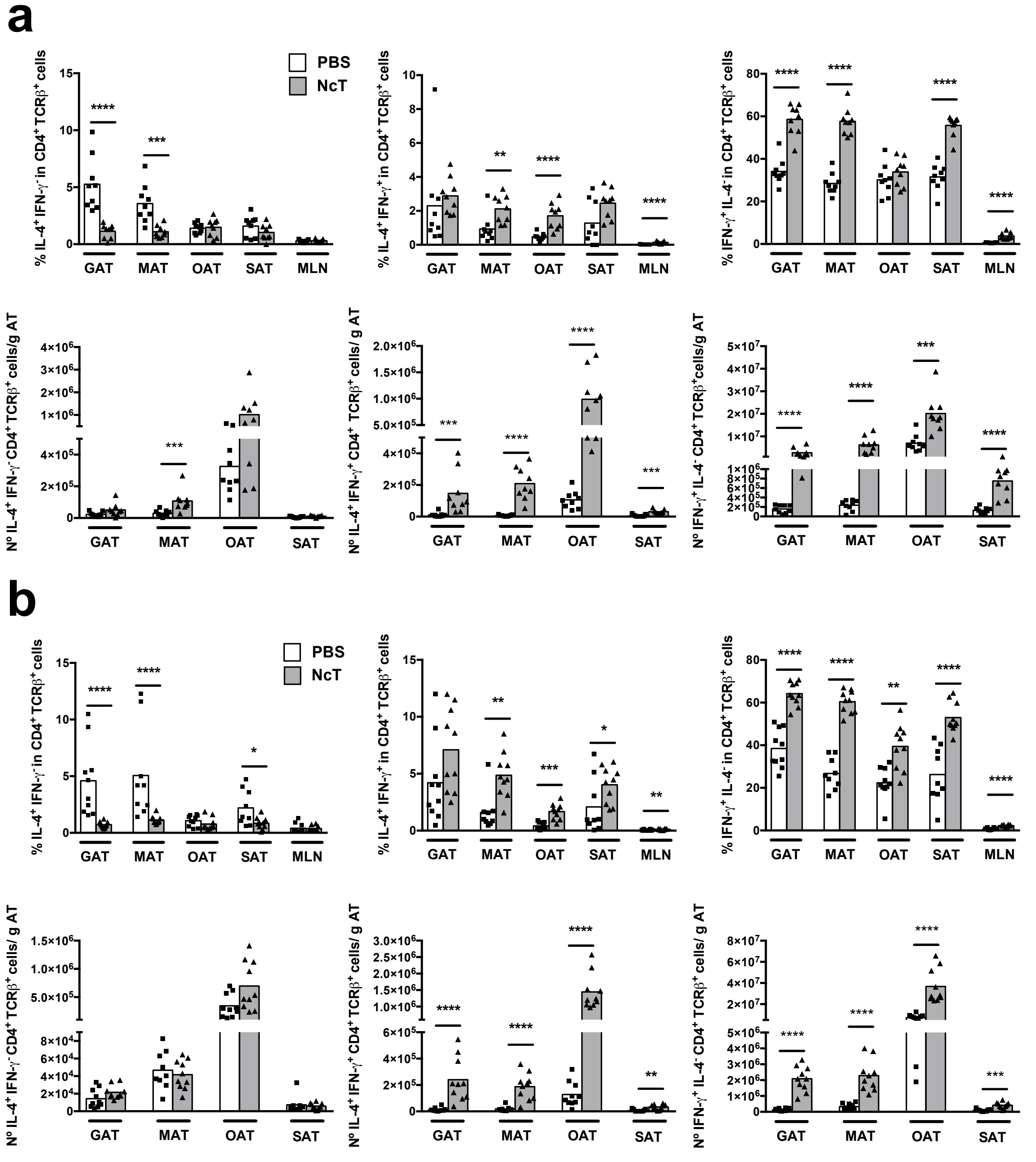

Frequency of IL-4^{+}IFN-γ^{-}CD4^{+}, IL-4^{+}IFN-γ^{+}CD4^{+} and IL-4^{-}IFN-γ^{+}CD4^{+} cells on total CD4^{+}TCRβ^{+}TCR$\gamma\delta^{-}$NK1.1^{-} cells and respective numbers per gram of adipose tissue in the gonadal, mesenteric, omental and subcutaneous adipose tissue (GAT, MAT, OAT and SAT, respectively) **(a)** 7 days or **(b)** 21 days after intraperitoneal challenge of wild-type mice with 1×10^{7} *N. caninum* tachyzoites (NcT) or PBS, as indicated. The frequencies of the same cellular populations in mesenteric lymph nodes (MLN) are also presented. Each symbol represents an individual mouse. Bars represent means of 9 mice per group pooled from 3 independent experiments. Statistically significant differences between different experimental groups are indicated (Mann-Whitney U test, **P* < 0.05; ***P* ≤ 0.01; ****P* ≤ 0.001; *****P* ≤ 0.0001).
